# An analysis of One Health timeliness metrics across multisectoral public health emergencies in Uganda

**DOI:** 10.1038/s43856-025-00893-9

**Published:** 2025-05-22

**Authors:** Jane K. Fieldhouse, Lydia Nakiire, Joshua Kayiwa, Ali Mirzazadeh, Claire D. Brindis, Ashley Mitchell, Jaime Sepulveda, Issa Makumbi, Alex Riolexus Ario, Elizabeth Fair, Sarah Gallalee, Herbert Isabirye, Musa Sekamatte, Brian H. Bird, Woutrina Smith, Angel Desai, Jonna A. K. Mazet, Mohammed Lamorde

**Affiliations:** 1 One Health Institute, University of California, Davis, CA USA; 2 Institute for Global Health Sciences, University of California, San Francisco, CA USA; 3 Office of Grand Challenges, University of California, Davis, CA USA; 4 Public Health Emergency Operations Centre, Ministry of Health Uganda, Kampala, Uganda; 5 Infectious Diseases Institute, Makerere University, Kampala, Uganda; 6 Philip R. Lee Institute for Health Policy Studies, University of California, San Francisco, CA USA; 7 Uganda National Institute of Public Health, Ministry of Health, Kampala, Uganda; 8 Division of Pulmonary and Critical Care Medicine, Department of Medicine, University of California, San Francisco, CA USA; 9 National One Health Platform, Ministry of Health Uganda, Kampala, Uganda

**Keywords:** Infectious diseases, Epidemiology

## Abstract

**Background:**

Timeliness metrics offer countries a framework by which to assess and optimize speed in outbreak detection and response times. This study analyses the One Health timeliness metrics for multisectoral public health emergencies in Uganda to identify and explore factors influencing outbreak performance.

**Methods:**

We compiled a database of outbreak events in Uganda occurring between 2018-2022 and involving the human, animal, plant, and environmental sectors. Outbreak milestone dates were extracted from reports to calculate timeliness metrics, which were analyzed using proportional hazards regression models. Concurrently, we conducted Key Informant Interviews to explore factors affecting detection and response timeliness.

**Results:**

Integrated analyses of timeliness metrics from 81 outbreaks and expert interviews reveal that the greatest predictors of improved timeliness are frequent past experience with similar disease outbreaks and whether an outbreak is a viral hemorrhagic fever due to heightened perceived threat and pre-existing preparedness measures. Other factors, including diagnostic and laboratory considerations and contextual influences, such as One Health collaborations, are also described as relevant to timeliness.

**Conclusions:**

To complement positive timeliness trends in Uganda, disease-agnostic investments in outbreak preparedness and response efforts will facilitate the ability of health systems to rapidly detect and respond to all outbreaks, irrespective of the pathogen.

## Abstract (web summary)

Fieldhouse et al. assess outbreak timeliness during health events in Uganda occurring between 2018-2022 and involving human, animal, plant, and environmental sectors. Detection and response times are strongest for diseases Uganda has previous experience with and for outbreaks of viral hemorrhagic fevers due to heighted perceived threat.

## Plain language summary

Rapid detection and response to disease outbreaks helps prevent their spread into larger, more harmful epidemics or pandemics. This study evaluates how quickly teams in Uganda detect and address disease outbreaks using a “One Health” approach, which integrates the health of humans, animals, plants, and the environment. By analyzing key dates of outbreaks between 2018 and 2022 and interviewing response experts, we compare detection and response times across different outbreak types. Findings reveal that while teams respond quickly to known diseases, novel diseases that the health system is unfamiliar with take longer to detect and address. Enhanced coordination between animal, human, and environmental health sectors could help prevent future pandemics by ensuring all outbreaks, regardless of type, are identified and controlled before widespread disease transmission occurs.

## Introduction

As the global Public Health Emergency declaration for the COVID-19 pandemic ends, the world once again risks perpetuating a cycle of panic followed by neglect for pandemic preparedness^1,2^. Given the probability of future extreme epidemics increasing in frequency^3^, efforts to bolster national and global preparedness for and performance during outbreaks remain more crucial than ever, to detect and respond rapidly to outbreaks, thereby averting future pandemics.

Estimates of increased intensity and frequency of epidemics in the future are largely attributed to the heightened risk of disease emergence from animal reservoirs associated with climate variability, change in land use, and loss of biodiversity^4,5^. Globally, experts recognize that a coordinated and integrated One Health approach, which emphasizes that the health of humans, animals, and our ecosystems are interdependent, is optimal and necessary to tackle such complex and interdisciplinary global challenges.

The Quadripartite^6^, comprised of the Food and Agriculture Organization of the United Nations, the United Nations Environment Programme, the World Health Organization (WHO), and the World Organisation for Animal Health, have issued an urgent call to action to strengthen collaboration and commitment to One Health, including enhanced intersectoral health governance and the implementation of One Health strategies to prevent pandemics and health threats at their source^7^.

To strategically inform policies and activities aimed at preventing pandemics, countries must first be able to objectively evaluate past and present performance to identify strengths and weaknesses in the outbreak landscape. Faster performance in outbreak detection and response allows collaborators a greater window of opportunity to slow or prevent disease spread, ideally translating to lives saved and a reduced socioeconomic toll associated with protracted outbreaks. Timeliness metrics, an objective measure of the time between key outbreak milestones, have been proposed as a tool to quantitatively assess detection and response times in order to optimize future responses^8,9^.

Several timeliness metrics frameworks have been proposed, including the 7-1-7 approach developed by Resolve to Save Lives, targeting detection of an infectious disease outbreak within 7 days, notification of relevant public health authorities within 1 day, and initiating a response within 7 additional days^10^.

**Fig. 1:**
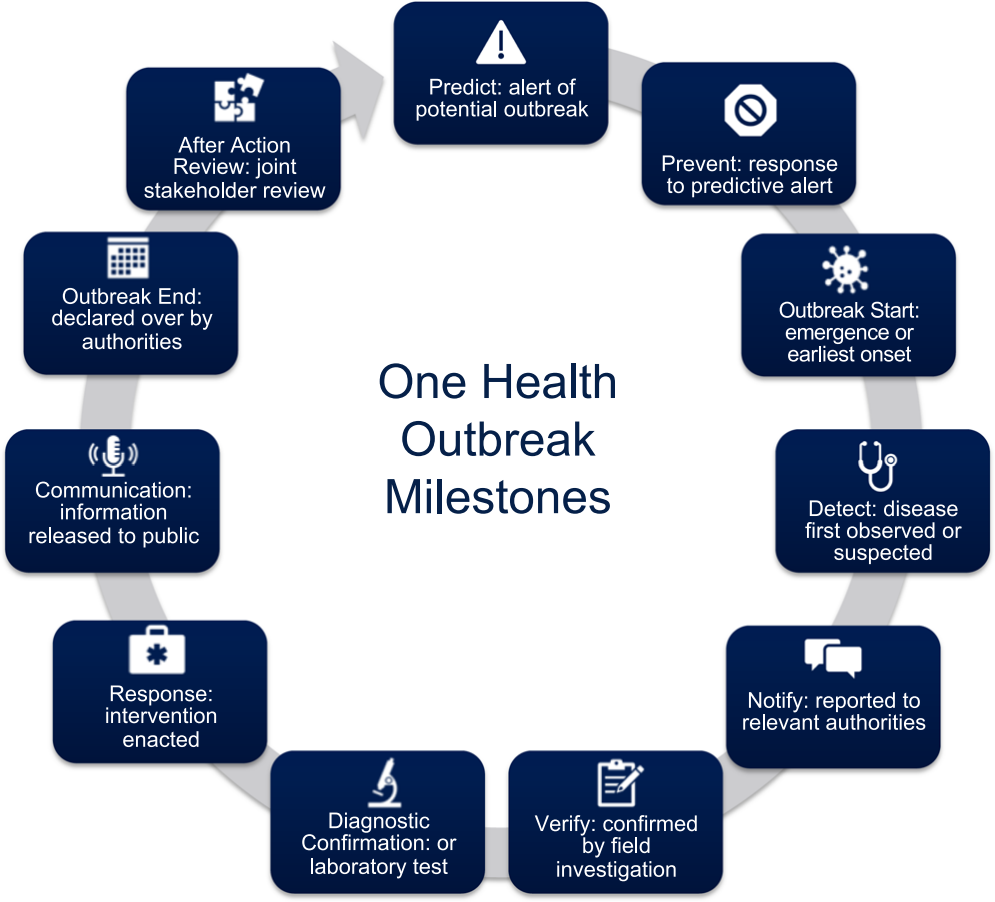
One Health outbreak milestones. Milestones defined by the Salzburg Global Seminar^8^. Timeliness metrics are calculated as the time in days between any two respective milestones. Milestones do not necessarily occur in this order or for every type of health event.

Building upon the metrics analyzed under the 7-1-7 approach, the One Health timeliness metrics have also been proposed as a framework similarly designed to assess performance in timeliness between 11 key outbreak milestones (Fig. 1).

The One Health timeliness framework, which emphasizes cross-sectoral coordination and community engagement, seeks to analyze metrics related to predictive alerts of outbreaks and preventive responses, when possible. Given assessments of priority pathogens routinely rank zoonotic and vector-borne diseases (as well as “Disease X”, which represents a currently unknown pathogen), as posing the greatest risk for pandemics and epidemics^11^, a truly integrated One Health approach is optimal to ensure that epidemic policy action, is transdisciplinary and unified. This approach includes monitoring and evaluation of environmental predictors (e.g., unusually heavy or unseasonal rainfall), potentially signaling amplified opportunities (e.g., intensified mosquito activity) for an outbreak in animal or human populations.

While analyses have demonstrated timeliness metrics as a useful tool for identifying where performance is fast and where there are lags in detection and response times^8,9,12,13^, these findings leave countries to speculate as to why bottlenecks occur and what factors promote speed. This study therefore seeks to use a mixed methods approach to explore timeliness during multisectoral outbreaks in Uganda, a country prone to outbreaks of diseases with epidemic and pandemic potential and one of the six countries in which the 7-1-7 targets have been successfully piloted^14^. Findings reveal strong timeliness performance, particularly for diseases Uganda has had past experience with and for outbreaks of viral hemorrhagic fevers due to the perceived high-threat of these pathogens. Timeliness is slowest for outbreaks of unknown or novel pathogens, with informants highlighting factors such as One Health collaborations, resource availability and allocation, and interoperability of reporting channels as influential to performance.

## Methods

We conducted a convergent mixed methods study with quantitative and qualitative data collection and analysis taking place concurrently in Uganda^15^. This study was conducted in parallel to the study described by Fieldhouse et al.^16^, which reported on the feasibility and utility of implementing timeliness metrics as a tool to assess and inform outbreak preparedness. This manuscript describes analyses of timeliness metrics during multisectoral outbreaks in Uganda, focusing on factors influencing speed during an outbreak.

### Quantitative methods

Collaborators from Makerere University’s Infectious Diseases Institute, Uganda’s Public Health Emergency Operations Centre (PHEOC), and the University of California (at Davis and San Francisco) developed a database of One Health outbreak milestones for public health events prompting activation of Uganda’s PHEOC between January 2018 and December 2022^16^. Outbreak events, which were organized in Microsoft Excel, were compared against a list of all PHEOC activations to check for completeness. Events had to meet Uganda’s Integrated Disease Surveillance and Response (IDSR) definition of an outbreak^17^. Additionally, events had to warrant multisectoral coordination by Uganda’s National One Health Platform, established in 2016, due to involvement of two or more of the One Health sectors (i.e., animals, humans, plants, or the environment). Diseases arising from only a human reservoir were therefore excluded from our database, as were natural disasters and activations for disease preparedness activities. Though it met our inclusion criteria, we additionally excluded COVID-19 from our analysis of timeliness data because the pandemic was an outlier in duration, scope, geographic spread, and response. Uganda’s response to the pandemic was, however, objectively fast, with preventive action (e.g., public gatherings suspended, quarantines enforced for travelers arriving in Uganda, etc.,) taken three days before the first case was detected.

The earliest date reported for each One Health milestone (Fig. 1) was extracted from original outbreak investigation reports, Situation Reports, Spot Reports, and other formal reports, which were compiled from PHEOC electronic records. Detailed definitions of each milestone are provided in Supplementary Table 1. To ensure maximum completeness of milestone dates, we additionally conducted a targeted literature review of outbreaks in Uganda (Supplementary Methods). Databases included reports from the U.S. Centers for Disease Control and Prevention’s Morbidity and Mortality Weekly Reports (MMWR)^18^, the World Health Organization’s Disease Outbreak News (DON) reports^19^, the World Organization for Animal Health (formerly OIE) Information System reports^20^, and the International Society for Infectious Diseases’ Program for Monitoring Emerging Diseases (ProMED) posts^21^. We also conducted a literature review of outbreaks reported in peer-reviewed journals published on the PubMed database between January 1, 2018 and December 31, 2022. Reports of outbreaks in Uganda for which the PHEOC was activated were reviewed for milestone dates. In instances of conflicting dates, we deferred to those in reports from the Government of Uganda.

In addition to extracting milestone dates (a day of a month of a year), a study team member (JKF) captured the following variables: the district(s) and region(s) in which the outbreak occurred; if the outbreak had crossed borders to or from another country; if Uganda had experience with similar outbreaks in the past either at the district or national level and, if so, the relative frequency of the occurrence in the country; transmission route and pathogen type; the surveillance method by which the outbreak was detected; and if the outbreak was an IDSR priority disease, condition, or event^17^. For the *Notify* milestone, we also made note of which authority was notified on the date recorded.

### Statistics and reproducibility

QGIS Version 3.12.3 (QGIS Geographic Information System, Open Source Geospatial Foundation Project) was used to map outbreaks by district. Statistical analyses were conducted in STATA version 16.0 (StataCorp, College Station, TX). Descriptive statistics of the outbreak characteristics were generated, along with the median time in days and interquartile ranges (IQR) between all respective milestones. Metrics were stratified by report year, region, disease, pathogen type, transmission route, surveillance type, One Health sectors involved, and whether the outbreak affected multiple countries, was a prioritized zoonotic disease^22^, or was a viral hemorrhagic fever (a group of febrile illnesses associated with high rates of morbidity and mortality). Outbreaks spanning multiple regions were categorized and analyzed by the region where the outbreak began, which is where the earliest milestone actions were most likely to have occurred. Outbreaks of suspected illness were categorized and analyzed by presumed etiology. We also stratified by frequency of past experience with a similar outbreak at the country level, categorized as “frequent” (PHEOC activation >10 in the past decade), “infrequent” (PHEOC activation ≤10 in the past decade), or “unknown” for outbreaks of undiagnosed illnesses.

Univariable and multivariable Cox proportional hazards regression analyses were conducted to assess changes in speed over time between two respective milestones. For outbreaks in which the predictor milestone (e.g., *Outbreak Start*) and outcome of interest milestone (e.g., *Detect*) occurred on the same date, we adjusted the second milestone to 0.3 days, or the equivalent of 8 hours. Based on the outcome of interest, missing dates were imputed based on the logic of subsequent milestone dates. For example, missing *Detect* dates were imputed with the next available milestone date (e.g., date of notification, verification, diagnostic confirmation, etc.) given the assumption that detection must have occurred for the subsequent milestones to transpire. Missing *Diagnostic Confirmation* dates were imputed using the *Public Communication* date if the outbreak reports indicated a confirmed diagnosis had occurred at some point in time. Missing *Response* dates were also imputed using *Public Communication* dates, and *End* dates were not imputed given no *After Action Review* dates were available for these missing dates.

Predictor variables included outbreak report year, relative frequency of experience with similar outbreaks, and whether the event was an outbreak of a viral hemorrhagic fever. Multicollinearity was assessed using a variance inflation factor (VIF) test. Survival curves were plotted to evaluate for proportional hazards assumptions. Findings are reported as estimated hazard ratios with 95% Confidence Intervals (CI). We used proportional hazards models after checking the potential violations of the proportional hazards assumption using log-minus-log survival plots and scaled Schoenfeld residual statistics^23^. All predictors satisfied the proportional hazards assumption via graphical and test evaluation.

### Qualitative methods

Purposive sampling methods were used to ensure perspectives of informants from different One Health sectors and levels of the health system. A study team member contacted 23 potential informants directly via email from a list of participants previously engaged in 7-1-7 implementation at national and subnational levels (Supplementary Methods). Ten (10) experts provided written consent and agreed to participate in 30- to 45-min recorded interviews. No compensation was provided.

Interviews were conducted by a trained researcher either in-person or remotely, per the interviewee’s preference. Interview questions were open-ended, and a semi-structured interview guide was used to explore factors affecting outbreak detection, investigation, or response timeliness. Participants were broadly asked to describe the latest outbreak they were involved in investigating or responding to, after which we asked: “What kinds of challenges can investigators encounter when responding to or reporting on outbreaks?” and “Are there any factors that contribute to more successful or more expedient responses to outbreaks?”.

Interviews were analyzed using a framework analysis^24,25^. This approach was chosen in order to produce a more structured form of a thematic analysis; though both framework analysis and thematic analysis involve systematic coding and theme development, framework analysis extends this by organizing data into a structured matrix to enable cross-case comparisons^26^. The stepwise analysis was performed by two study members (JKF and AM) who, following familiarization with the transcripts, identified a thematic framework based on the open-coding of four transcripts using Dedoose Version 9.0.90 (SocioCultural Research Consultants, Los Angeles, CA, 2023). Over a series of meetings, the study team compared, revised, and re-coded all transcripts, using a cyclical approach to coding. Codes were then categorized and charted into the framework table in Excel. Iterative exploration was conducted in the visual collaboration platform, Miro (2023), to explore big-picture relationships and guide further explanations.

### Inclusion and ethics declarations

This study received approval by the Infectious Diseases Institute Research and Ethics Committee in Uganda (#IDIREC REF 077/2022), and the Uganda National Council for Science and Technology (registration number HS2255ES). The study was deemed exempt by the University of California, Davis Institutional Review Board (IRB ID 1778303-1). This research was conducted by a diverse and multidisciplinary team of collaborative researchers at various career stages. Efforts were made to ensure the research design and findings are inclusive and representative of broader communities.

### Reporting summary

Further information on research design is available in the Nature Portfolio Reporting Summary linked to this article.

## Results

### Quantitative findings

**Fig. 2:**
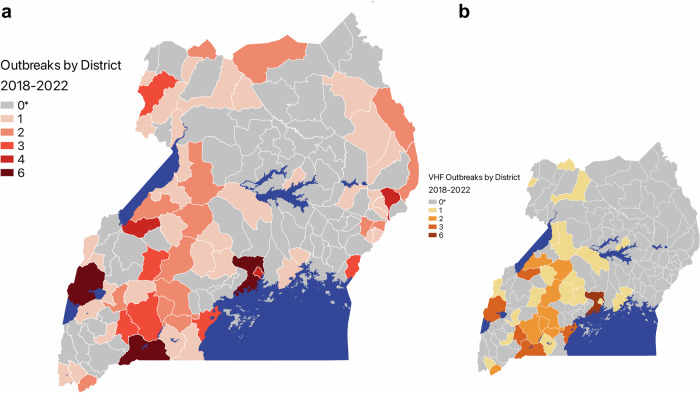
Map of outbreak events in Uganda occurring 2018-2022. The distribution of all 81 outbreaks included in our timeliness metrics analysis (**a**) and the distribution of the 31 viral hemorrhagic fever outbreaks (**b**) across the 135 districts of Uganda. *No outbreaks in these districts were included in our analysis; however, outbreaks could have occurred in these districts between 2018 and 2022 that did not prompt activation of the PHEOC or were excluded from our database for not meeting inclusion criteria.

The PHEOC was activated 302 times for 282 epidemiologically distinct public health emergencies between 2018 and 2022. Of these events, 129 were outbreaks meeting our inclusion criteria, and complete documentation was available for 82 events (64%). After removing COVID-19 from the dataset, 81 outbreaks remained in the analysis (Fig. 2). Though most dates included in the analysis were captured from the PHEOC-provided reports, we included twenty-three milestone dates found through our literature review, including ten dates from peer-reviewed publications found on PubMed, six from ProMED, four from MMWR, and three from the WHO.

The most frequent outbreak included in the dataset was cholera, constituting 28% (*n* = 22) of events, followed by anthrax (18%; *n* = 14), Rift Valley fever (15%, *n* = 12), and Crimean-Congo hemorrhagic fever (15%, *n* = 12); (Supplementary Data 1). Missing dates for reported outbreak milestones are outlined in the Supplementary Information file (Supplementary Table 2).

Our analysis of timeliness metrics found an overall median time of 3 days (IQR 1–5) between outbreak *Start* and date of *Detection*, 2 days (IQR 0–9) between *Detection* and *Notification*, and 5 days (IQR 2–16) between *Detection* and *Diagnostic Confirmation* (Supplementary Data 1). The median time from *Detection* to *Outbreak End* was 57 days (IQR 37–95). A complete list of overall timeliness metrics is available in the Supplementary Information file (Supplementary Table 3), along with details on imputation for the proportional hazards models.

Results from the multivariable Cox proportional hazards model found significant improvements over time in timeliness for the *Start* to *End* interval (Table 1). Hazard ratios demonstrated the greatest improvements in 2019 and 2021, with smaller but sustained gains in 2020 and 2022 compared to 2018. Timeliness also improved from 2018 to 2019 for the *Detect* to *Diagnostic* interval (HR 2.58, 95% CI 1.12–5.96) and the *Detect* to *End* interval (HR 3.30, 95% CI 1.47–7.41). There were statistically significant reductions in hazard ratios for the *Detect* and *Respond* interval in 2020 and 2022, (HR 0.18, 95% CI 0.07–0.48 in 2020 and HR 0.29, 95% CI 0.12–0.74 in 2022), suggesting slower responses in these two years compared to 2018.

**Table 1:** Multivariable Cox proportional hazards regression analysis Hazard Ratios (HR) with report year, past experience, and VHF as predictor variables for select timeliness metrics intervals

	Start to detect (*n* = 55)		Detect to diagnostic (*n* = 52)		Detect to respond (*n* = 52)		Start to end (*n* = 50)		Detect to end (*n* = 67)	
Predictors	*N* (%)	Hazard ratio per year (95% CI)	*N* (%)	Hazard ratio per year (95% CI)	*N* (%)	Hazard ratio per year (95% CI)	*N* (%)	Hazard ratio per year (95% CI)	*N* (%)	Hazard ratio per year (95% CI)
Report year										
2018	14 (25)	Ref.	13 (25)	Ref.	14 (27)	Ref.	10 (20)	Ref.	15 (22)	Ref.
2019	10 (18)	1.87 (0.74–4.70)	7 (13)	2.58 (1.12–5.96)^a^	10 (19)	0.56 (0.25–1.27)	10 (20)	10.51 (3.11–35.61)^a^	14 (21)	3.30 (1.47– 7.41)^a^
2020	12 (22)	1.33 (0.56–3.12)	13 (25)	0.58 (0.26–1.31)	12 (23)	0.18 (0.07–0.48)^a^	11 (22)	7.03 (2.17–22.83)^a^	14 (21)	1.67 (0.76– 3.68)
2021	6 (11)	1.41 (0.50– 3.94)	7 (13)	1.26 (0.47–3.39)	5 (10)	0.39 (0.13–1.18)	6 (12)	10.83 (2.71–43.29)^a^	9 (13)	2.69 (0.99–7.34)
2022	13 (24)	1.27 (0.45– 3.59)	12 (23)	0.55 (0.21–1.42)	11 (21)	0.29 (0.12–0.74)^a^	13 (26)	5.64 (1.62–19.64)^a^	15 (22)	1.30 (0.58– 2.92)
Experience										
Unknown	1 (2)	0.0	0	–	1 (2)	1.00 (0.11–9.3)	1 (2)	2.17 (0.21–22.60)	2 (3)	19.20 (2.6–141.60)
Infrequent	8 (15)	Ref.	8 (15)	Ref.	9 (17)	Ref.	8 (16)	Ref.	9 (13)	Ref.
Frequent	46 (84)	1.45 (0.59–3.59)	44 (85)	2.96 (1.33–6.59)^a^	42 (81)	2.13 (0.86–5.27)	41 (82)	11.27 (3.39–37.44)^a^	56 (84)	5.57 (2.17–14.27)^a^
VHF										
No	29 (53)	Ref.	33 (55)	Ref.	32 (62)	Ref.	26 (52)	Ref.	41 (61)	Ref.
Yes	26 (47)	0.44 (0.22–0.91)^a^	27 (45)	2.73 (1.37–5.46)^a^	20 (38)	1.33 (0.70–2.53)	24 (48)	1.34 (0.62–2.89)	26 (39)	2.67 (1.33–5.35)^a^

*CI* Confidence Interval, *NB* A Hazard Ratio of >1 indicates faster times compared to the reference (i.e., shorter time to the milestone outcome).

^a^Statistically significant findings (α = 0.05).

Cox proportional hazards models found that, across most timeliness intervals, having frequent past experience with similar diseases and being a viral hemorrhagic fever (VHF) outbreak were the greatest predictors of improved timeliness (Table 1). VIF test values for all regression models were ≤2.35, suggesting any correlation between past experience and VHFs was moderate and unlikely to result in unreliable regression findings.

**Fig. 3:**
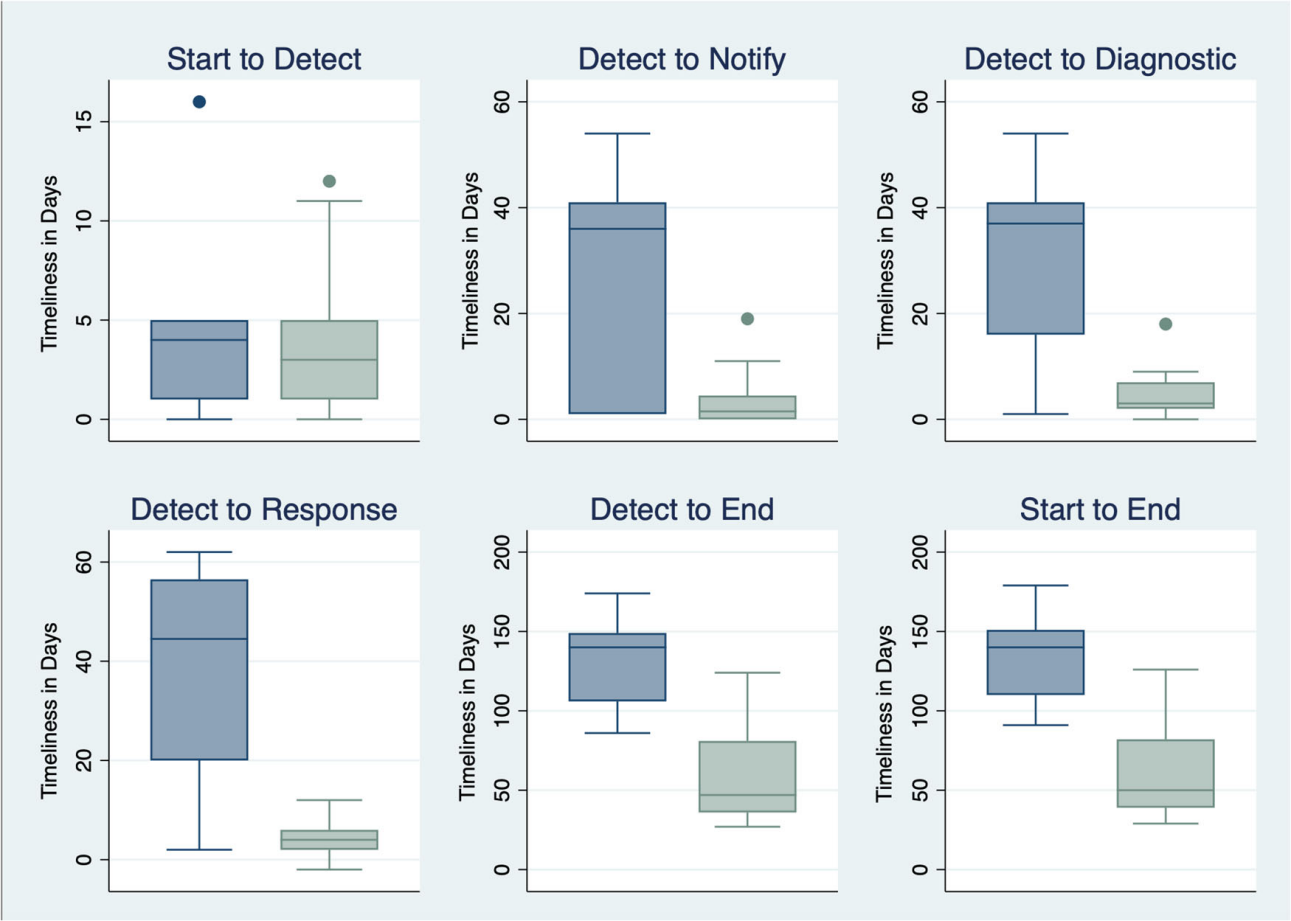
Box plots of median times in days between select One Health milestones, by outbreak type frequency. Box plots show median time in days between two respective milestones, by outbreaks that have occurred infrequently (≤10 outbreaks prompting PHEOC activation in the past decade) versus frequently (>10 outbreaks prompting PHEOC activation in the past decade). Extreme outliers are not shown. Unknown frequency of five undiagnosed outbreaks excluded. Box-plot elements include: the center line indicating the median; box limits indicating the upper and lower quartiles; and whiskers representing ±1.5 × interquartile range, with outlier points indicated outside the whiskers. Source data for this figure can be found in Supplementary Data 3.

For most intervals, timeliness was faster for frequent outbreaks (>10 PHEOC activations in the past decade) compared to infrequent outbreaks (≤10 PHEOC activations in the past decade) (Fig. 3). This finding remained true regardless of the outbreak year or if the outbreak was a VHF, with hazards models found timeliness increased 196% between *Detect* and *Diagnostic* for outbreaks categorized as occurring frequently compared to infrequently (HR2.96, 95% CI 1.33–6.59). Though the confidence intervals were fairly wide, we found a 1027% improvement in timeliness from *Start* to *End* for frequent outbreaks (HR 11.27, 95% CI 3.39–37.44) and a 457% improvement in timeliness between *Detect* and *End* milestones (HR 5.57, 95% CI 1.17–14.27) compared to infrequent ones.

Compared to outbreaks of non-VHFs, outbreaks of VHFs had a timeliness improvement of 173% (HR 2.73, 95% CI 1.37–5.46) from the date of detection to diagnostic confirmation and an improvement of 167% (HR 2.67, 95% CI 1.33–5.35) from the date of detection to the outbreak end. However, timeliness from *Start* to *Detect* was slower for outbreaks of VHFs (median time 4 days, IQR 3–6) compared to non-VHFs (median time 2 days, IQR 1–4, a timeliness decrease of 56% (HR 0.44, 95% CI 0.21–0.90).

In an analysis by disease, median days from *Start* to *Detect*, *Detect* to *Diagnostic*, and *Detect* to *End* were longest for yellow fever compared to all other diseases, except for the *Notify* to *Diagnostic Confirmation* metric. The median time for the latter metric is 0 days (*n* = 3) but in each instance, the earliest date of notification on record was the Uganda Virus Research Institute (UVRI) notifying various responsible parties of the positive diagnostic confirmation.

### Qualitative findings

Ten (10) of 23 invited experts agreed to participate in a recorded interview. Participants, only one of whom identified as female, represented seven institutions at the regional, national, and international levels of the health system in Uganda (Supplementary Table 4). Four of the informants had backgrounds in epidemiology (all levels), two in veterinary science (national level), two in laboratory science (all levels), and two in health information analytics (national and international).

As detailed in Supplementary Data 2, three broad themes emerged from our framework analysis of factors influencing timeliness: 1) factors related to the outbreak event itself; 2); contextual influences on the outbreak, and 3) considerations related to the experiences of frontline workers. Informants described how, at the national level, experience with similar outbreaks in the past may improve outbreak response. Furthermore, disease-specific biases, such as perceived threat level of the outbreak, may impact various intervals of timeliness (e.g., responses for Marburg and Ebola are mounted more quickly due to perceived severity of the disease). Additionally, informants characterized One Health collaborations as an enabler to detection and notification, while also citing delays attributable to the disproportionate allocation of resources to the human health sector. Other bottlenecks and enablers, which were described interchangeably depending on their absence or presence, included availability of resources or resource mobilization, transport of specimens, interoperability of reporting channels, community knowledge or trust in public health system, and challenges specific to laboratory and diagnostic capacity (Supplementary Figs. 1 and 2). As described by one informant, even knowing that mpox was a public health emergency in 2022, it took a month for Uganda to establish diagnostic capacity to conduct surveillance for the virus.

## Discussion

In contrast with studies of timeliness metrics at regional or global levels, findings from this body of work reveal unique country-level strengths and challenges for Uganda during outbreak events. Country-specific analyses can be incredibly important for tracking and improving preparedness and response efforts, while additionally providing insights for other countries in the region planning for similar events. The findings are also useful more broadly because of the detailed data collected by Uganda and the mixed methods approach, which allows for specific insights not often achievable with other datasets.

Analyses of timeliness during multisectoral outbreaks in Uganda occurring between 2018 and 2022 suggest that overall national performance in detection and response time is relatively strong. Uganda performed faster than the 7-1-7 targets for two intervals (i.e., outbreak start to detection and detection to response)^14^, and compared to timeliness observed during outbreaks occurring between 2017 and 2019 in WHO AFRO member states, Uganda had shorter median times to events for nearly all intervals (Supplementary Table 5)^12^.

Uganda’s strong performance times were particularly pronounced for outbreaks of diseases with which the country has had previous experience, a quantitative finding supported key informant interviews. Given Uganda has seen repeat outbreaks of several diseases, sometimes even on an annual basis, this finding points to a positive or improved timeliness trend for familiar outbreak types in Uganda.

A convergence of qualitative and quantitative findings additionally illustrates that the perceived threat of VHFs results in heightened preparation for outbreaks of certain diseases (i.e., the development of risk communication materials for Marburg and Ebola virus disease [EVD]), as well as a faster overall response times. This finding is supported by our previous analysis of frequency of One Health milestone reporting, which found that more timeliness data were reported for VHFs compared to outbreaks of non-VHFs^16^. Preparedness for and rapid response to diseases of a high threat ultimately contributed to the successful control of the 2022 outbreak of Sudan EVD, which was brought under control before it grew into an event of severe enough spread to warrant declaration of a Public Health Emergency of International Concern^27^.

While timeliness during outbreaks of VHFs may avert morbidity and mortality, perhaps even prevent a pandemic, the comparative lags in response to diseases considered of a lesser threat as well as diseases that countries have less experience with, could limit pandemic preparedness. Non-agnostic approaches may lead to longer delays between key milestones during outbreaks of novel, unknown diseases, including *Disease X*. Findings from this study provide empirical support for leveraging pre-existing collaborations across sectors and networks to improve response for diseases, regardless of the causative pathogen.

Outbreak response in Uganda was also demonstrated as critically dependent on specific outbreak capacity. Examples include laboratory diagnostics, as highlighted by the observed length of outbreak metrics and key informant discussions of yellow fever (Table 1, Supplementary Data 1, 2). The sub-themes of diagnostic and laboratory considerations and existing infrastructure and health system structures, as well as most other sub-themes emerging from our qualitative analysis, aligned closely with findings from previous studies of barriers to and enablers of outbreak detection and response^14,28–30^. For instance, qualitative work among COVID-19 response teams in Papua New Guinea found similar response barriers, including laboratory considerations (e.g., cold chain availability) and inadequate human resources, while identifying community engagement as a key enabler to testing^28^. A systematic review of the literature conducted by Swaan et al.^29^ also identified reporter motivation, availability of resources, and laboratory considerations^29^.

Based on their qualitative study conducted among experts responding to the zoonotic Middle East respiratory syndrome epidemic, van Roode et al.^30^ offer a global perspective on systemic challenges on data sharing during zoonotic outbreaks, many of which overlap with our findings at the national level in Uganda^30^. Key issues identified include suboptimal collaboration, specifically citing coordination challenges across One Health disciplines and sectors, as well as barriers related to technical preparedness such as laboratory infrastructure and capacity. One of the core dilemmas identified related to the dichotomy of needing international assistance and coordination for outbreaks versus recognizing and respecting the sovereignty of countries^30^. Our findings reflect this dilemma at the national level, with informants describing that international influences (including funding) may sway Uganda’s priorities, thus impeding what should be a wholly nationally driven prioritization process.

van Roode et al. also found that funding and investment disparities affected multisectoral outbreak response^30^. Slower release of funding to non-human sectors generates bureaucratic bottlenecks, which in turn inhibit responder’s ability to mount a truly coordinated and collaborative One Health response to outbreaks. In some instances, illness or die-off events first observed within animal populations may even serve as a predictive alert of an outbreak of human illness. For example, in 2012, staff trained in wildlife disease surveillance through the USAID PREDICT project discovered six howler monkeys dead near a wildlife sanctuary in Bolivia^31^. Post-mortem diagnostic tests (RT-PCR) confirmed infection by flavivirus, later confirmed as yellow fever virus. The MoH was immediately notified of the findings, enabling prompt implementation of public health prevention measures such as human vaccination campaigns, education and outreach, and mosquito control measures. Consequently, no human cases of yellow fever occurred during this outbreak^32^.

Furthermore, inequities in resource allocation to different health sectors contributes to perceptions of power imbalances across disciplines, as described by informants. These power imbalances are detrimental to efforts to build trust across sectors, which can be devastating to efforts to work across the necessary collaborators required to prevent and control outbreaks. (Supplementary Note).

Discrepancies between when funds and supplies are released to human versus animal health sectors illustrates a qualitative finding that could not be explored in depth through our quantitative analyses. This limitation is due in large part to our sampling strategy for our database, which was linked to the PHEOC under the Ministry of Health. Consequently, our data is biased toward human diseases as opposed to those diseases affecting exclusively the animal, environmental, and plant sectors.

Additionally, we recognize that while COVID-19 was removed from our dataset, the timeframe of outbreaks included in our analysis captures outbreaks occurring against the backdrop of the pandemic. Despite Uganda’s yearly improvements in *Start* to *End* timeliness, observed gains in speed for several intervals in 2019 were followed by declines in 2020 and 2022, perhaps reflecting resource and operational challenges posed by the pandemic^33^. Statistically significant reductions in *Detect* to *Respond* intervals in 2020 and 2022, for example, may have been due to disruptions in human and financial resources which were redirected to respond to COVID-19^34^. Given the hypothesis that we might see faster timeliness following the start of the pandemic due to heightened vigilance and surveillance efforts, these findings underscore the importance of building resilient detection and response systems capable of maintaining performance for outbreak events even during a global health crisis.

Notably, there is also the possibility of biased quantitative findings, given that 36% of outbreaks events occurring during the study timeframe were excluded due to missing documentation. Regarding incomplete data for the 81 outbreaks analyzed here, a secondary study of these timeliness data conducted by a team at Resolve to Save Lives conducted a missingness analysis based on patterns of missing dates. (Kim, S. et al., submitted *BMJ Global Health*) The analysis found a predominantly random pattern of missing dates across most disease categories, except for undiagnosed illnesses. While this randomness would suggest minimal risk, we cannot exclude the possibility of bias due to missing data. We must also recognize that our assumptions underlying the dates used for imputation in the proportional hazards models may have also led to biased findings.

The limitations of our database underscore broader shortcomings of timeliness metrics as a tool: quantitative snapshots of timeliness risk missing key findings such as the perceived importance of a collaborative One Health approach in increasing speed during outbreaks. Comprehensive insight into the complex processes during outbreaks requires complementary qualitative work, a step which could logically take place during an *After-Action Review* meeting, when multidisciplinary partners have the opportunity to convene and discuss strengths and challenges encountered. Establishing a systems approach in analyzing bottlenecks and other problems versus blaming of any one individual or sector, is key in establishing the level of trust necessary to constructively review negative, as well as positive factors.

These analyses also must consider that in some instances, timeliness metrics may simply reflect epidemiological characteristics of the disease-causing pathogen, rather than the performance of responders^29^. For example, foodborne illnesses may naturally have a shorter duration from start to end than VHFs or diseases with longer incubation periods. Furthermore, timeliness in determining the mode of exposure will depend on the causative pathogen and type of outbreak (i.e., a more prolonged process in foodborne outbreaks) and will influence subsequent metrics. Our models may not fully capture such nuances, including within-pathogen variability or contextual factors like outbreak setting. Recent implementation of the 7-1-7 global timeliness targets to tuberculosis found field staff recommended an adaptation of these metrics to ‘3-5-7’ based on the epidemiology and screening process for the bacterium^35^.

The type of surveillance used to detect the outbreak will also influence timeliness metrics, particularly for indicator-based surveillance depending on proximity to a health facility. As this study did not capture exact geographic coordinates of the outbreak start, we were unable to analyze how location (i.e., rurality) impacted detection times.

We additionally recognize that timeliness metrics may be subject to measurement bias due to interpretation of outbreak milestone dates. Milestone date extraction was conducted by one study team member from the University of California^16^. This study member built upon previous experience interpreting One Health milestone dates based on a study of thousands of outbreak reports, for which exercises were conducted across three investigators to validate interpretation of dates^36^.

While useful for identifying trends in timeliness, the One Health timeliness metrics alone are not a panacea and must be utilized alongside other pandemic preparedness and prevention tools to inform epidemic and pandemic policy action. Furthermore, timeliness metrics are premised on an assumption that increased speed between intervals results in improved outbreak outcomes, such as reduced morbidity and mortality in human and animal populations. In our comparison of timeliness metrics calculated for this study in Uganda versus timeliness calculated across WHO AFRO by Impouma et al.^12^ we saw that despite having faster overall *Start* to *Detect* and *Detect* to *Notify* times in 2018, Uganda had a longer overall *Start* to *End* interval. Additional analyses should explore this assumption that increased speed between all intervals translates to improved outbreak outcomes, as well as other possible explanations for the observed phenomena, such as differences in definitions of outbreak end. Analyses might also explore if certain timeliness metrics, including those with predictive alerts and preventive action, are more or less influential on improved outcomes.

## Conclusions

As teams of experts worldwide continue to develop innovative strategies to confront our pandemic era, collaborators must remain cognizant of the need for disease-agnostic outbreak preparedness activities, such that health systems are resilient when faced with an outbreak of a novel disease. Moreover, efforts to achieve truly integrated One Health collaborations for outbreak detection and response may ultimately improve timeliness during outbreaks involving multiple health sectors, underscoring the need for tailored policies and strategies to address specific challenges identified.

Continued tracking and comparisons of timeliness during outbreaks involving versus not involving the human sector are warranted, as are studies of timeliness during outbreaks with predictive alerts, which would inherently necessitate collaborations across One Health sectors to detect and to respond with preventive measures. For all timeliness metrics analyses, a systems approach to evaluate and then monitor successes and challenges for quality improvement will prevent the onus from being placed on any one sector or group of responders.

In countries where it has been a problem to assess both progress and whether timeliness continues to lag because of slow release of funding, we recommend tracking when funds are released for outbreak investigation. Toward this objective, we echo a recommendation made by an informant in this study, that the Quadrapartite pool response funds for any agencies that needs to respond to an outbreak, regardless of health sector. Ongoing activities to build trust, ensure equitable access to funding, and establish integrated channels for communication, reporting, and data sharing will better position countries to mount a unified front against epidemics. In addition to building trust between frontline workers from different health sectors, experts have described that fostering trust with communities is paramount to successful control of outbreaks.

Once partnerships and trust are established between key One Health collaborators, becoming routine and expected for all outbreaks, it will be less of a shock to the health system when synergistic relationships are needed to address larger-impact emergencies and the emergence of *Disease X*.

## Supplementary information

Supplementary Information

Description of Additional Supplementary files

Supplementary Data 1

Supplementary Data 2

Supplementary Data 3

Reporting Summary

## Data Availability

Supplementary Data 1 contains timeliness metrics data (median time in days between two respective milestones along with interquartile ranges) stratified by predictor variables. Supplementary Data 2 outlines qualitative themes and sub-themes identified through the study analysis, along with illustrative quotes from key informants. In addition to data in Supplementary Data files 1 and 2, the complete dataset generated and analyzed for the study is available from the corresponding author, JKF, upon request. The timeliness metrics data informing Fig. 3 can be found in Supplementary Data 3.

## References

[1] World Health Organization. *Statement on the fifteenth meeting of the IHR (2005) Emergency Committee on the COVID-19 pandemic* (WHO, 2023).

[2] Yamey, G., Schäferhoff, M., McDade, K. K. & Mao W. *Preparing for pandemics such as coronavirus—will we ever break the vicious cycle of panic and neglect?* (Future Development Brookings Institution, 2020).

[3] Marani, M., Katul, G. G., Pan, W. K. & Parolari, A. J. Intensity and frequency of extreme novel epidemics. *Proc. Natl Acad. Sci. USA.* **118**, e2105482118 (2021). 34426498 10.1073/pnas.2105482118 PMC8536331

[4] Daszak, P., Cunningham, A. A. & Hyatt, A. D. Anthropogenic environmental change and the emergence of infectious diseases in wildlife. *Acta Trop.* **78**, 103–116 (2001). 11230820 10.1016/s0001-706x(00)00179-0

[5] Gibb, R. et al. Zoonotic host diversity increases in human-dominated ecosystems. *Nature* **584**, 398–402 (2020). 32759999 10.1038/s41586-020-2562-8

[6] Joint Tripartite (FAO OIE WHO) and UNEP Statement. *Tripartite and UNEP support OHHLEP’s definition of “One Health”*. Available from: https://www.who.int/news/item/01-12-2021-tripartite-and-unep-support-ohhlep-s-definition-of-one-health (2021).

[7] World Health Organization. Quadripartite call to action for One Health for a safer world. Rome/Paris/Geneva/Nairobi. Available from: https://www.who.int/news/item/27-03-2023-quadripartite-call-to-action-for-one-health-for-a-safer-world (2023).

[8] Crawley, A. W., Divi, N. & Smolinski, M. S. Using timeliness metrics to track progress and identify gaps in disease surveillance. *Health Secur.* **19**, 309–317 (2021). 33891487 10.1089/hs.2020.0139

[9] Dos S Ribeiro, C. et al. A framework for measuring timeliness in the outbreak response path: lessons learned from the Middle East respiratory syndrome (MERS) epidemic, September 2012 to January 2019. *Eur. Surveill.* **27**, 2101064 (2022). 10.2807/1560-7917.ES.2022.27.48.2101064 PMC9716647 36695460

[10] Frieden, T. R., Lee, C. T., Bochner, A. F., Buissonnière, M. & McClelland, A. 7-1-7: an organising principle, target, and accountability metric to make the world safer from pandemics. *Lancet* **398**, 638–640 (2021). 34242563 10.1016/S0140-6736(21)01250-2 PMC9636000

[11] Ukoaka, B. M. et al. Updated WHO list of emerging pathogens for a potential future pandemic: Implications for public health and global preparedness. *Infez. Med.* **32**, 463–477 (2024). 39660154 10.53854/liim-3204-5 PMC11627490

[12] Impouma, B. et al. Measuring timeliness of outbreak response in the World Health Organization African Region, 2017-2019. *Emerg. Infect. Dis.* **26**, 2555–2564 (2020). 33079032 10.3201/eid2611.191766 PMC7588517

[13] Chan, E. H. et al. Global capacity for emerging infectious disease detection. *Proc. Natl Acad. Sci. USA* **107**, 21701–21706 (2010). 21115835 10.1073/pnas.1006219107 PMC3003006

[14] Bochner, A. F. et al. Implementation of the 7-1-7 target for detection, notification, and response to public health threats in five countries: a retrospective, observational study. *Lancet Glob. Health* **11**, e871–e879 (2023). 37060911 10.1016/S2214-109X(23)00133-X PMC10156425

[15] Creswell, J. W. & Clark, V. L. P. *Designing and conducting mixed methods research* (Sage publications, 2017).

[16] Fieldhouse, J. et al. How feasible or useful are timeliness metrics as a tool to optimise One Health outbreak responses? *BMJ Glob. Health* **9**, e013615 (2024). 38991578 10.1136/bmjgh-2023-013615 PMC11268058

[17] Republic of Uganda Ministry of Health. *National technical guidelines for integrated disease surveillance and response*, 3rd ed. (Republic of Uganda Ministry of Health, 2021).

[18] Centers for Disease Control and Prevention. *Morbidity and Mortality Weekly Report: MMWR*. U.S. Dept. of Health, Education, and Welfare, Public Health Service, Center for Disease Control. Available from: https://www.cdc.gov/mmwr/.

[19] World Health Organization. *Disease Outbreak News (DONs)*. Available from: https://www.who.int/emergencies/disease-outbreak-news.

[20] World Organisation for Animal Health. OIE World Animal Health Information System. Available from: https://wahis.woah.org/#/home

[21] Chase, V. ProMED: a global early warning system for disease. *Environ. Health Perspect.* **104**, 699 (1996). 8841752 10.1289/ehp.104-1469400 PMC1469400

[22] Sekamatte, M. et al. Multisectoral prioritization of zoonotic diseases in Uganda, 2017: a One Health perspective. *PLoS One* **13**, e0196799 (2018). 29715287 10.1371/journal.pone.0196799 PMC5929520

[23] Kleinbaum, D. G. & Klein, M. *Survival analysis a self-learning text*, 3rd ed. (Springer; 2012).

[24] Ritchie, J. & Spencer, L. Qualitative data analysis for applied policy research. In: Bryman, A. & Burgess, R., editors. *Analyzing qualitative data* (Routledge, 1994) 173–194.

[25] Gale, N. K., Heath, G., Cameron, E., Rashid, S. & Redwood, S. Using the framework method for the analysis of qualitative data in multi-disciplinary health research. *BMC Med. Res. Methodol.* **13**, 117 (2013). 24047204 10.1186/1471-2288-13-117 PMC3848812

[26] Braun, V. & Clarke, V. Using thematic analysis in psychology. *Qual. Res. Psychol.* **3**, 77–101 (2006).

[27] Aceng, J. R. et al. Continental concerted efforts to control the seventh outbreak of Ebola Virus disease in Uganda: The first 90 days of the response. *J. Public Health Afr.* **14**, 2735 (2023). 37881727 10.4081/jphia.2023.2735 PMC10594597

[28] Smaghi, B. S. et al. Barriers and enablers experienced by health care workers in swabbing for COVID-19 in Papua New Guinea: A multi-methods cross-sectional study. *Int. J. Infect. Dis.* **110**, S17–S24 (2021). 33991678 10.1016/j.ijid.2021.04.077 PMC8116122

[29] Swaan, C., van den Broek, A., Kretzschmar, M. & Richardus, J. H. Timeliness of notification systems for infectious diseases: a systematic literature review. *PLOS ONE* **13**, e0198845 (2018). 29902216 10.1371/journal.pone.0198845 PMC6002046

[30] van Roode, M. Y. et al. Six dilemmas for stakeholders inherently affecting data sharing during a zoonotic (re-)emerging infectious disease outbreak response. *BMC Infect. Dis.* **24**, 185 (2024). 38347527 10.1186/s12879-024-09054-0 PMC10863217

[31] Kelly T. R. et al. Implementing One Health approaches to confront emerging and re-emerging zoonotic disease threats: lessons from PREDICT. *One Health Outlook* **2**, 1 (2020). 10.1186/s42522-019-0007-9 PMC7149069 33824944

[32] Uhart, M. et al. A ‘One Health’ Approach to Predict Emerging Zoonoses in the Amazon. 10.13140/RG.2.1.3549.1609 (2012).

[33] The World Bank. COVID-19 Economic Crisis and Recovery Development Policy Financing (P173906) (PGD203). Retrieved from https://documents1.worldbank.org/curated/en/609321593741824637/pdf/Uganda-COVID-19-Economic-Crisis-and-Recovery-Development-Policy-Financing.pdf (2020).

[34] Republic of Uganda Ministry of Health. Corona Virus Disease - 2019 (COVID-19) Preparedness and Response Plan March 2020-June 2021. Kampala, Uganda Retrieved from https://covid19.gou.go.ug/uploads/document_repository/authors/ministry_of_health/document/ (2020).

[35] Harries, A. D. et al. Applying ‘timeliness’ to the screening and prevention of TB in household contacts of pulmonary TB patients. *IJTLD Open.* **1**, 59–62 (2024). 38966694 10.5588/ijtldopen.23.0615 PMC11221595

[36] Fieldhouse, J. K. et al. One Health timeliness metrics to track and evaluate outbreak response reporting: a scoping review. *eClinicalMedicine* **53**, 101620 (2022). 36097540 10.1016/j.eclinm.2022.101620 PMC9463558

