# Supplementary material for: An analysis of One Health timeliness metrics across multisectoral public health emergencies in Uganda
Source: Commun Med (Lond). 2025 May 22;5:192. doi: 10.1038/s43856-025-00893-9 (PMC12098913; doi:10.1038/s43856-025-00893-9)
Supplement: Supplementary file 1 — Supplementary Information [file 43856_2025_893_MOESM1_ESM.pdf]

Supplementary Information

An analysis of One Health timeliness metrics across multisectoral public health emergencies in Uganda

Jane K. Fieldhouse[1,2,3]*, Lydia Nakiire[4,5], Joshua Kayiwa[4], Ali Mirzazadeh[2], Claire D. Brindis[2,6], Ashley Mitchell[2], Jaime Sepulveda[2], Issa Makumbi[4], Alex Riolexus Ario[7], Elizabeth Fair[2,8], Sarah Gallalee[2], Herbert Isabirye[4], Musa Sekamatte[9], Brian H. Bird[1], Woutrina Smith[1], Angel Desai[1,3], Jonna A.K. Mazet[1,3]*, Mohammed Lamorde[5]

1 One Health Institute, University of California, Davis, California, USA
2 Institute for Global Health Sciences, University of California, San Francisco, California, USA
3 Office of Grand Challenges, University of California, Davis, California, USA
4 Public Health Emergency Operations Centre, Ministry of Health Uganda, Kampala, Uganda
5 Infectious Diseases Institute, Makerere University, Kampala, Uganda
6 Philip R. Lee Institute for Health Policy Studies, University of California, San Francisco
7 Uganda National Institute of Public Health, Ministry of Health, Kampala, Uganda
8 Division of Pulmonary and Critical Care Medicine, Department of Medicine, University of California, San Francisco, California, USA
9 National One Health Platform, Ministry of Health Uganda, Kampala, Uganda

*Corresponding Authors:

Jane Fieldhouse:
Email: jkfieldhouse@ucdavis.edu
Tel (US): +1-650-609-7372

Jonna Mazet:
Email: jkmazet@ucdavis.edu
Tel (US): +1- 530 752 2065

Supplementary Methods

Quantitative

Targeted Literature Review

We conducted a review of online archives of outbreak reports published between January 2018 and December 2022. The peer-reviewed literature search was conducted on PubMed. The search string used was ("disease outbreaks"[MeSH Terms] AND "Uganda"[Title/Abstract]) AND (2018:2022[pdat]). Publication titles and abstracts were screened to determine if the event described an outbreak meeting our inclusion criteria, described below. Articles meeting these criteria were then read in full and dates for each milestone described were extracted.

The archives of the U.S. Centers for Disease Control and Prevention's Morbidity and Mortality Weekly Reports (MMWR), the World Health Organization's Disease Outbreak News (DON) reports, the World Organization for Animal Health (formerly OIE) Information System reports, and the International Society for Infectious Diseases' Program for Monitoring Emerging Diseases (ProMED) posts, were reviewed for any outbreak occurring in Uganda between 2018 and 2022. Of note, this review was conducted before ProMED limited access to archives of posts dating beyond 30 days. These reports were organized in Microsoft Excel along with URL links to the reports. Outbreaks were organized by event (i.e., multiple reports on the same epidemiologically linked event) and cross-checked against a complete list of all Public Health Emergency Operations Centre (PHEOC) activations in Uganda during the time period. Reports were then reviewed for inclusion and exclusion criteria.

Inclusion and Exclusion Criteria

The inclusion and exclusion criteria were the same across the peer-reviewed literature and grey literature published by the health organizations and agencies. Per our inclusion criteria, events had to prompt Uganda's PHEOC activation, which is determined on an event-by-event basis by the Director General of Health Services based on the existing guidelines on Integrated Diseases Surveillance and Response (IDSR) thresholds. We included both activations occurring at the alert and response level, which depend on the perceived severity and magnitude of the event. Events had to meet Uganda's IDSR definition of an outbreak as "the occurrence of more cases than expected in a defined geographic area or time." [2] Lastly, outbreaks had to involve two or more One Health sectors, namely humans, animals, plants, or the environment. Therefore, diseases arising from a human reservoir and circulating exclusively within human populations were excluded. This included surges in tuberculosis or measles cases.

Imputation for Proportional Hazards models

As described, missing dates were imputed in the Cox proportional hazards models based on the logic of subsequent milestone dates. A total of three dates were imputed for the *Detect* milestone using the date of notification (n =1) and date of verification (n=2). Eight (8) dates for *Diagnostic Confirmation* were imputed, all using the date of public communication. Five (5) dates were imputed for the *Response* milestone, also using the date that public communication occurred.

Qualitative

Key Informant Inclusion and Exclusion Criteria

Informants must be stakeholders in the Uganda public health response landscape or have related experience in researching outbreak events in Uganda. Informants had to have knowledge about and be involved in or have previously been involved in an aspect of outbreak detection or response, including but not limited to outbreak surveillance, epidemiologic investigations, laboratory or diagnostic testing, data sharing, outbreak reporting, and public health policy.

Informants were excluded if they were unavailable or unwilling to participate in interviews during the data collection period of the study. Interviews were exclusively conducted in English and therefore informants who did not speak English would have been excluded from this study; however, all informants approached were fluent and therefore no one was excluded based on this criterion.

Below are the detailed definitions of the eleven One Health outbreak milestones, adapted with additional details from their original publication by the Salzburg Global Seminar.[1]

Supplemental Table 1. One Health Outbreak Milestone Definition

**Milestone**	**Definition**
Predict	The date of a valid alert of a potential health threat that could prompt a potential outbreak (e.g., increased rainfall leading to increased mosquito activity; a landslide churning up soil and potentially increasing the risk of an anthrax outbreak).
Prevent	The date that enhanced surveillance or another intervention is initiated in response to a predictive alert (e.g., mosquito abatement or a mass vaccination).
Outbreak Start	The date of the earliest epidemiologically-linked symptom onset or death, in either humans or animals. Most often this date is identified retrospectively and may be estimated based on evidence during an outbreak investigation.
Detect	The date of symptom onset, death, or evidence of circulation in humans or animals that is first observed or suspected. Detection may occur at the community level or within the health system (e.g., health facility, laboratory, or surveillance) and will depend on what type of surveillance was used to detect the event.
Notify	The date the event is officially reported to relevant and responsible authorities. This may include cross-sector notification, as well as notification from local to national, or national to international levels.
Verify	The date of confirmation by field investigation or other valid method.
Diagnostic Confirmation	The date of diagnostic test or laboratory confirmation in an epidemiologically-linked human or animal.
Respond	The date of when an intervention was enacted in order to control or manage an outbreak, as initiated by the responsible authorities (e.g., quarantine, food recall, or vaccination campaign).
Public Communication	The date of the official release of information to the public from the responsible authority (e.g., press release or media appearance).
Outbreak End	The date when the outbreak is formally declared closed by a responsible authority.
After-Action Review	The date when a joint review of the outbreak occurred by relevant One Health authorities.

Supplementary Results

Quantitative
The literature search of outbreaks described above yielded 296 results. Based on a screening of article titles and abstracts, 44 publications met our inclusion criteria. Our review of the grey literature from organizations found 45 events documented by the International Society for Infectious Diseases' Program for Monitoring Emerging Diseases (ProMED) posts. We found seven events which met our outbreak criteria published in the World Health Organization's Disease Outbreak News (DON) reports, seven from the World Organization for Animal Health Information System reports, and six from the U.S. Centers for Disease Control and Prevention's Morbidity and Mortality Weekly Reports.

All of the reports identified through the literature review described outbreaks that prompted activation of PHEOC. Consequently, the majority of the milestone dates analyzed in this study were extracted from documentation provided by the PHEOC. However, from this literature review, we were able to find an additional 23 milestone dates for 14 of the outbreak events, which were missing from the original documentation provided by the PHEOC.

Supplemental Table 2. Reporting frequency of the One Health outbreak milestones for 81 analyzed outbreak events occurring in Uganda between 2018-2022

Milestone	Specific milestone date reported (%)	Milestone date mentioned, no date (%)	Milestone not mentioned at all (%)
Predict	11 (14)	8 (10)	62 (76)
Prevent	2 (2)	0 (0)	79 (98)
Outbreak Start	55 (68)	4 (5)	22 (27)
Detect	78 (96)	1 (1)	2 (2)
Notify	56 (69)	4 (5)	21(26)
Verify	63 (78)	10 (12)	8 (10)
Diagnostic*	54 (67)	21 (26)	3 (4)
Respond	51 (63)	24 (30)	6 (7)
Communication	35 (43)	28 (35)	18 (22)
Outbreak End	69 (85)	1 (1)	11 (14)
After Action Review	0 (0)	4 (5)	77 (95)

*Three (3) additional reports provided specific milestone dates for which diagnostic confirmation was unsuccessful, and etiology remained unconfirmed

Supplemental Table 3. Overall timeliness metrics for 81 outbreak events in Uganda occurring 2018-2022. Timeliness metrics defined as the median time in days between two outbreak milestones and Inter Quartile Ranges (IQR) where n = number of outbreak reports reporting specific date of both milestones. Milestones have been organized in sequential order, from left to right, recognizing that several milestones between *Detect* and *Communication* may not always occur in the exact order of events.

Milestone	Predict	Prevent	Start	Detect	Notify	Verify	Diagnostic	Respond	Communi-cation	End	Review
**Predict**		98<br>n=1	16 (4-42)<br>n=8	14 (7-60)<br>n= 11	10 (9-117)<br>n= 8	11 (9-61)<br>n= 7	15 (10-119)<br>n= 8	22 (12-59)<br>n= 7	17 (9-28)<br>n=7	82 (62-100)<br>n= 9	n=0
**Prevent**			215<br>n=1	215<br>n=1	216<br>n= 1	216<br>n= 1	216<br>n= 1	217<br>n= 1	216<br>n= 1	355<br>n= 1	n=0
**Start**				3 (1-5)<br>n=54	7 (3-15)<br>n=42	7 (3-17)<br>n=47	9 (6-23)<br>n=39	8 (5-15)<br>n=37	10 (4-20)<br>n=27	61 (43-110)<br>n=50	n=0
**Detect**					2 (0-9)<br>n=54	2 (1-7)<br>n=61	4 (2-13)<br>n=52	4 (2-9)<br>n=50	6 (3-9)<br>n=34	57 (37-95)<br>n=67	n=0
**Notify**						0 (0-3)<br>n=48	1 (0-4)<br>n=38	1 (0-5)<br>n=38	3 (1-7)<br>n=28	56 (36-95)<br>n=47	n=0
**Verify**							0 (-1-2)<br>n=48	1 (0-3)<br>n=46	2 (0-4)<br>n=31	47 (35-86)<br>n=54	n=0
**Diagnostic**								1 (-1-3)<br>n=41	2 (0-3)<br>n=26	49 (35-83)<br>n=49	n=0
**Respond**									0 (-1-4)<br>n=30	52 (33-87)<br>n=47	n=0
**Communication**										64 (34-89)<br>n=33	n=0
**End**											n=0

Qualitative

Supplemental Table 4: Description of key informant occupations and level of health systems represented

KII Participant Occupation	Health System Level
Health Information Analyst	International
Virologist, Researcher	International / National
Veterinarian	National
Public Health Information Analyst	National
Veterinarian, Epidemiologist	National
Medical Epidemiologist	National
Public Health Officer	National
Epidemiologist	Regional / National
Environmental Health Scientist, Epidemiologist	Regional
Medical Lab Scientific Officer	Regional

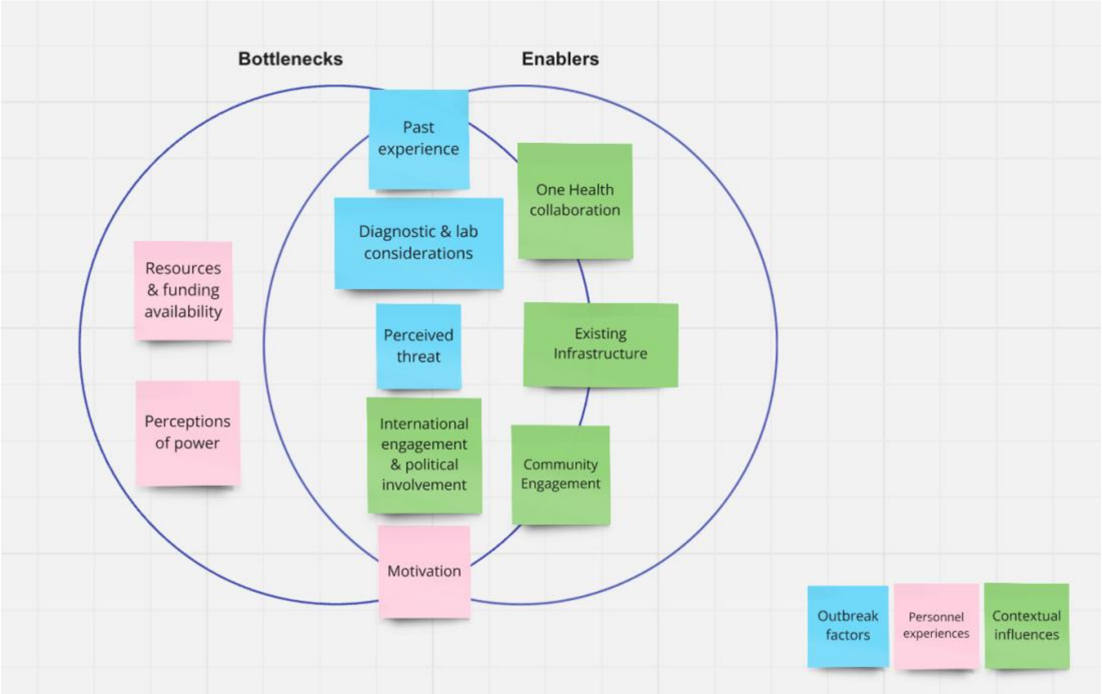

Supplemental Figure 1. Visual exploration of how themes and sub-themes are discussed by participants as either bottlenecks, enablers, or both, to the process of identifying and responding to outbreaks.

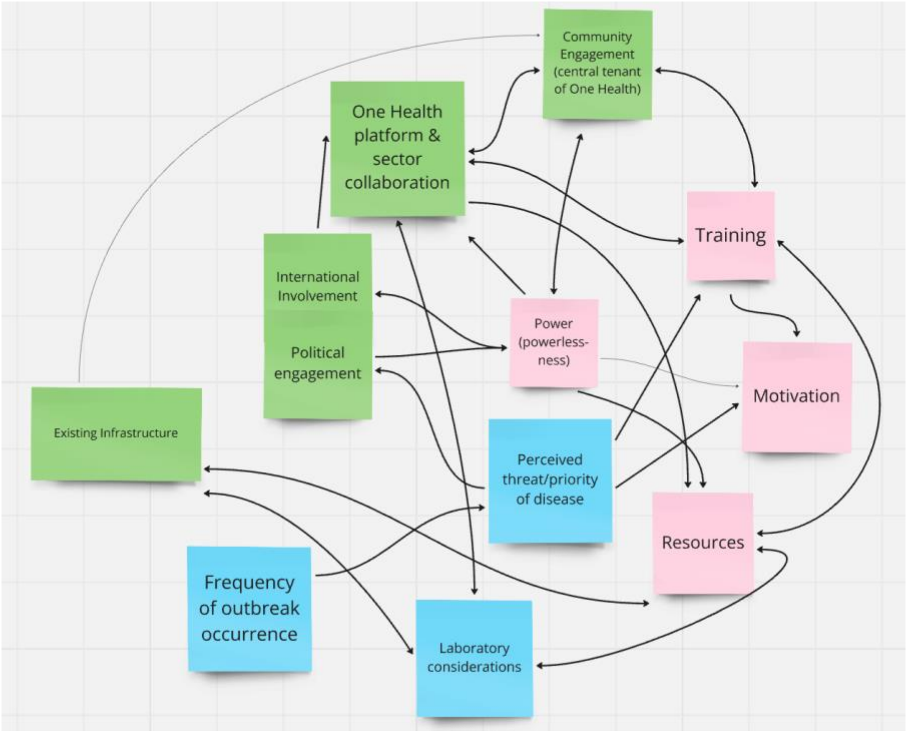

Supplemental Figure 2. Visual exploration of the relationships between sub-themes described by key informants.

Supplemental Table 5: Comparison of outbreak timeliness metrics in Uganda and WHO AFRO region (Impouma et al. 2020)[3] from 2018 and 2019 for three timeliness intervals

	Start* to *Detect		***Detect* to *Notify***		***Start* to *End***	
	**Uganda**	**WHO AFRO***	**Uganda**	**WHO AFRO***	**Uganda**	**WHO AFRO***
**2018** Median days (IQR) N Reports (%†)	4 (2-4) n=14 (78)	7 (1-27) n = 62 (61)	2 (0-3) n=12 (67)	3 (0-14) n=83 (82)	118 (64-130) n=10 (56)	67 (25–144) n=72 [3]
**2019** Median days (IQR) N Reports (%†)	1 (0-3) n=9 (60)	4 (1–11) n=47 (54)	2 (0-5) n=6 (40)	4 (1-9) n=62 [3]	44 (29-57) n=10 (67)	45 (22–90) n=67 (77)

*Data on WHO AFRO region from 41 out of 47 member states. Reference: Impouma B, Roelens M, Williams GS, et al. Measuring Timeliness of Outbreak Response in the World Health Organization African Region, 2017-2019. *Emerg Infect Dis* 2020; **26**(11): 2555-64.

†Total reports for 2018 in Uganda n = 18, WHO AFRO n=101; total reports for 2019 in Uganda n= 15, WHO AFRO n= 87.

Supplementary Note

Lessons from Yellow Fever in Uganda

In this study, the observed timeliness from outbreak detection to end was longer for outbreaks of yellow fever than any other disease, with a median time of 135 days (n=4, IQR 114-162) versus 53 days for other diseases (n=63, IQR 36-90). Despite it being a VHF, timeliness decreased 69% between outbreaks of other diseases and outbreaks of YF, regardless of outbreak year (HR 0.31, 95% CI 0.11-0.87).

Our analysis included five outbreaks of YF occurring between 2018 and 2022. Timeliness from outbreak start to detection was similar to that of other diseases; however, timeliness from outbreak detection to diagnostic confirmation was 41 days (n=3, IQR 16-54) compared to a median time of 4 days for all other diseases (n=49, IQR 2-8). Diagnostic confirmation of YF is a particularly long process, and not all laboratories will have the many supplies required. There are also very few validated commercial assays for the virus on the market, a challenge compounded by specific timing requirements for sample collection following disease onset, complex diagnostic testing algorithms for unvaccinated versus vaccinated people, and a host of other laboratory challenges.[4]

Beyond diagnostic considerations, YF may be particularly challenging for responders due to the relative novelty of the virus in the outbreak landscape in Uganda. In November of 2010, Uganda experienced the first detected outbreak of yellow fever since the 1970s, when surveillance activities slowed until the Integrated Disease Surveillance and Response system was introduced in 2000.[5] YF outbreaks have occurred sporadically in Uganda since 2010, until more recently in 2019, when outbreaks began to occur on an annual basis, with one to two outbreaks each year.

The 2010 outbreak was the largest of YF the country had experienced to-date. However, due to the unfamiliarity with the virus, given several decades had passed since the last outbreak of YF, responders were not experienced in detecting and bringing the outbreak under control. Given the challenging case definition, the 2010 outbreak was initially believed to be an outbreak of plague. With no treatment (antivirals) for yellow fever, vaccination and other prevention measures such as vector control are essential to bringing outbreaks under control. Unfortunately, the response was hampered because of need for a diagnosis in order to then determine which pool of funds to release for investigation. This bottleneck highlights the importance of our recommendation to quickly release funds for the investigation of a suspect outbreak, whether or not the etiology has been confirmed yet.

Supplemental References:
1. Salzburg Global Seminar. New Timeliness Metrics Seek to Improve Pandemic Preparedness. 2020. Accessed: https://www.salzburgglobal.org/news/latest-news/article/new-timeliness-metrics-seek-to-improve-pandemic-preparedness
2. Republic of Uganda Ministry of Health. National Technical Guidelines for Integrated Disease Surveillance and Response. Third ed. Kampala, Uganda; 2021.
3. Impouma B, Roelens M, Williams GS, et al. Measuring Timeliness of Outbreak Response in the World Health Organization African Region, 2017-2019. *Emerg Infect Dis* 2020; **26**(11): 2555-64.
4. World Health Organization. Yellow fever laboratory diagnostic testing in Africa. Geneva, Switzerland; 2016.
5. Wamala JF, Malimbo M, Okot CL, et al. Epidemiological and laboratory characterization of a yellow fever outbreak in northern Uganda, October 2010–January 2011. *International Journal of Infectious Diseases* 2012; **16**^{3}: e536-e42.
