# Supplementary material for: An analysis of One Health timeliness metrics across multisectoral public health emergencies in Uganda
Source: Commun Med (Lond). 2025 May 22;5:192. doi: 10.1038/s43856-025-00893-9 (PMC12098913; doi:10.1038/s43856-025-00893-9)
Supplement: Supplementary file 2 — Description of Additional Supplementary files [file 43856_2025_893_MOESM2_ESM.pdf]

Description of Additional Supplementary files

File name: Supplementary Data 1

The source data for timeliness metrics (median time in days between two respective milestones along with interquartile ranges) stratified by predictor variables.

File name: Supplementary Data 2

The source data for qualitative themes and sub-themes identified through the study analysis, along with illustrative quotes from key informants.

In addition complete dataset generated and analyzed for the study is available from the corresponding author, JKF, upon request.

File name: Supplementary Data 3

The source data for timeliness metrics informing Figure 3.
