# Supplementary material for: An analysis of One Health timeliness metrics across multisectoral public health emergencies in Uganda
Source: Commun Med (Lond). 2025 May 22;5:192. doi: 10.1038/s43856-025-00893-9 (PMC12098913; doi:10.1038/s43856-025-00893-9)
Supplement: Supplementary file 6 — Reporting Summary [file 43856_2025_893_MOESM6_ESM.pdf]

nature portfolio

Corresponding author(s): Jane K. Fieldhouse

Last updated by author(s): Mar 28, 2025

Reporting Summary

Nature Portfolio wishes to improve the reproducibility of the work that we publish. This form provides structure for consistency and transparency in reporting. For further information on Nature Portfolio policies, see our Editorial Policies and the Editorial Policy Checklist.

Statistics

For all statistical analyses, confirm that the following items are present in the figure legend, table legend, main text, or Methods section.

n/a	Confirmed	
☒	☐	The exact sample size (*n*) for each experimental group/condition, given as a discrete number and unit of measurement
☒	☐	A statement on whether measurements were taken from distinct samples or whether the same sample was measured repeatedly
☐	☒	The statistical test(s) used AND whether they are one- or two-sided<br>*Only common tests should be described solely by name; describe more complex techniques in the Methods section.*
☐	☒	A description of all covariates tested
☐	☒	A description of any assumptions or corrections, such as tests of normality and adjustment for multiple comparisons
☐	☒	A full description of the statistical parameters including central tendency (e.g. means) or other basic estimates (e.g. regression coefficient) AND variation (e.g. standard deviation) or associated estimates of uncertainty (e.g. confidence intervals)
☒	☐	For null hypothesis testing, the test statistic (e.g. *F*, *t*, *r*) with confidence intervals, effect sizes, degrees of freedom and *P* value noted<br>*Give P values as exact values whenever suitable.*
☒	☐	For Bayesian analysis, information on the choice of priors and Markov chain Monte Carlo settings
☒	☐	For hierarchical and complex designs, identification of the appropriate level for tests and full reporting of outcomes
☒	☐	Estimates of effect sizes (e.g. Cohen's *d*, Pearson's *r*), indicating how they were calculated

Our web collection on statistics for biologists contains articles on many of the points above.

Software and code

Policy information about availability of computer code

Data collection	Outbreak events were organized in Microsoft Excel Version 16.93.1.
Data analysis	QGIS Version 3.12.3 (QGIS Geographic Information System, Open Source Geospatial Foundation Project) was used to map outbreaks by district. Statistical analyses were conducted in STATA version 16.0 (StataCorp, College Station, TX).

For manuscripts utilizing custom algorithms or software that are central to the research but not yet described in published literature, software must be made available to editors and reviewers. We strongly encourage code deposition in a community repository (e.g. GitHub). See the Nature Portfolio guidelines for submitting code & software for further information.

Data

Policy information about availability of data

All manuscripts must include a data availability statement. This statement should provide the following information, where applicable:

- Accession codes, unique identifiers, or web links for publicly available datasets
- A description of any restrictions on data availability
- For clinical datasets or third party data, please ensure that the statement adheres to our policy

The dataset generated and analyzed for the study is available from the corresponding author, JKF, upon request.

Research involving human participants, their data, or biological material

Policy information about studies with human participants or human data. See also policy information about sex, gender (identity/presentation), and sexual orientation and race, ethnicity and racism.

Reporting on sex and gender	Gender of participants in the key informant interviews was reported (with one participant identifying as female) but no analyses based on gender were performed.
Reporting on race, ethnicity, or other socially relevant groupings	No data on participant race, ethnicity, or other socially relevant groupings were collected; instead, only information about their field of work and expertise,
Population characteristics	No data on participant age or sociodemographics were collected; instead, only information about their field of work and expertise.
Recruitment	Purposive sampling methods were used to recruit participants to participate in the interviews directly via email. We used a list of individuals who had previously participated in related outbreak metrics work at national and subnational levels given these experts were from diverse sectors and all key stakeholders in the health of humans, animals, and the environment in Uganda. We recognize that informants may therefore have a biased perspective of the utility of timeliness metrics, given that we recruited participants who had previously been invited to participate in related timeliness workshops. Key informants interviewed for this study also did not represent all levels of the health system. However, the study participants were heterogeneous across different sectors, and their perspectives may represent individuals better positioned to report on perceived strengths and limits of the framework given their first-hand experience.
Ethics oversight	This study received approval by the Infectious Diseases Institute Research and Ethics Committee in Uganda (#IDIREC REF 077/2022), and the Uganda National Council for Science and Technology (registration number HS2255ES). The study was deemed exempt by the University of California, Davis Institutional Review Board (IRB ID 1778303-1).

Note that full information on the approval of the study protocol must also be provided in the manuscript.

Field-specific reporting

Please select the one below that is the best fit for your research. If you are not sure, read the appropriate sections before making your selection.

☐ Life sciences ☒ Behavioural & social sciences ☐ Ecological, evolutionary & environmental sciences

For a reference copy of the document with all sections, see nature.com/documents/nr-reporting-summary-flat.pdf

Behavioural & social sciences study design

All studies must disclose on these points even when the disclosure is negative.

Study description	This was a convergent parallel mixed methods study with quantitative and qualitative data collection and analysis taking place concurrently.
Research sample	For the qualitative work, we conducted key informant interviews with professionals with experience and expertise working in outbreak investigations.
Sampling strategy	Purposive sampling was used for our qualitative research and interviews were conducted until we deemed that theoretical saturation was achieved. To do so, we iteratively reviewed responses from participants in the form of transcripts to evaluate if new themes or patterns were still emerging from new participants.
Data collection	Only the study participant and interviewer were present during Key Informant Interviews. Interviews were conducted either in person or via Zoom depending on the participant's preference. For interviews conducted via Zoom, we recorded audio using the Zoom platform, with a back up recording captured through the encrypted Apple Voice Memo application. For interviews conducted in person, we recorded the audio using the encrypted Apple Voice Memo application. For all interview types, paper and pen were used to take notes during the interview on a copy of the interview guide. Recordings were transcribed into Microsoft Word documents immediately following the interview conclusion, after which all audio recordings were destroyed (deleted).
Timing	Quantitative data was collected on outbreaks occurring between January 1, 2018 and December 31, 2022. Qualitative data was collected (interviews conducted) between June 22, 2022 and September 13, 2022.
Data exclusions	No qualitative data were excluded from the study. Of the quantitative data collected, we decided to exclude COVID-19 from our timeliness metrics analyses, not just because it was ongoing at the end of our study period, but also because the pandemic was an outlier in duration, scope, geographic spread, and response.
Non-participation	In total, we contacted 23 potential informants directly via email to invite them to interview; 11 initially agreed to an interview but one experienced technical difficulties connecting via Zoom for a remote interview and never responded to requests to re-schedule. Therefore, only 10 completed an interview with a study member.

Randomization	N/A

Reporting for specific materials, systems and methods

We require information from authors about some types of materials, experimental systems and methods used in many studies. Here, indicate whether each material, system or method listed is relevant to your study. If you are not sure if a list item applies to your research, read the appropriate section before selecting a response.

Materials & experimental systems

n/a	Involved in the study
☒	☐ Antibodies
☒	☐ Eukaryotic cell lines
☒	☐ Palaeontology and archaeology
☒	☐ Animals and other organisms
☒	☐ Clinical data
☒	☐ Dual use research of concern
☒	☐ Plants

Methods

n/a	Involved in the study
☒	☐ ChIP-seq
☒	☐ Flow cytometry
☒	☐ MRI-based neuroimaging

Plants

Seed stocks	N/A
Novel plant genotypes	N/A
Authentication	N/A
